# Primary Human Colon Epithelial Cells (pHCoEpiCs) Do Express the Shiga Toxin (Stx) Receptor Glycosphingolipids Gb3Cer and Gb4Cer and Are Largely Refractory but Not Resistant towards Stx

**DOI:** 10.3390/ijms221810002

**Published:** 2021-09-16

**Authors:** Johanna Detzner, Charlotte Püttmann, Gottfried Pohlentz, Hans-Ulrich Humpf, Alexander Mellmann, Helge Karch, Johannes Müthing

**Affiliations:** 1 Institute for Hygiene, University of Münster, 48149 Münster, Germany; Johanna.Detzner@ukmuenster.de (J.D.); lotte02021996@web.de (C.P.); pohlentz@uni-muenster.de (G.P.); Alexander.Mellmann@ukmuenster.de (A.M.); hkarch@uni-muenster.de (H.K.); 2 Institute for Food Chemistry, University of Münster, 48149 Münster, Germany; humpf@uni-muenster.de

**Keywords:** colon epithelial cells, Gb3Cer, Gb4Cer, glycosphingolipids, Stx1a, Stx2a, Stx2e

## Abstract

Shiga toxin (Stx) is released by enterohemorrhagic *Escherichia coli* (EHEC) into the human intestinal lumen and transferred across the colon epithelium to the circulation. Stx-mediated damage of human kidney and brain endothelial cells and renal epithelial cells is a renowned feature, while the sensitivity of the human colon epithelium towards Stx and the decoration with the Stx receptor glycosphingolipids (GSLs) globotriaosylceramide (Gb3Cer, Galα1-4Galβ1-4Glcβ1-1Cer) and globotetraosylceramide (Gb4Cer, GalNAcβ1-3Galα1-4Galβ1-4Glcβ1-1Cer) is a matter of debate. Structural analysis of the globo-series GSLs of serum-free cultivated primary human colon epithelial cells (pHCoEpiCs) revealed Gb4Cer as the major neutral GSL with Cer (d18:1, C16:0), Cer (d18:1, C22:1/C22:0) and Cer (d18:1, C24:2/C24:1) accompanied by minor Gb3Cer with Cer (d18:1, C16:0) and Cer (d18:1, C24:1) as the dominant lipoforms. Gb3Cer and Gb4Cer co-distributed with cholesterol and sphingomyelin to detergent-resistant membranes (DRMs) used as microdomain analogs. Exposure to increasing Stx concentrations indicated only a slight cell-damaging effect at the highest toxin concentration of 1 µg/mL for Stx1a and Stx2a, whereas a significant effect was detected for Stx2e. Considerable Stx refractiveness of pHCoEpiCs that correlated with the rather low cellular content of the high-affinity Stx-receptor Gb3Cer renders the human colon epithelium questionable as a major target of Stx1a and Stx2a.

## 1. Introduction

Among numerous gastrointestinal pathogens that cause severe gastroenteritis in humans enterohemorrhagic *Escherichia coli* (EHEC), the major subgroup of virulent Shiga toxin (Stx)-producing *E. coli* (STEC) can colonize the human gut mucosa [1,2], where EHEC are able to interact via attaching and effacing (A/E) lesions with the host colon epithelium. Such A/E lesions are characterized by intimate pathogen attachment to the apical surface of enterocytes and reorganization of the actin cytoskeleton beneath the adhered bacteria into pedestals leading to brush border and microvilli deterioration [3,4]. The locus of enterocyte effacement (LEE), a genome-inserted pathogenicity island, comprises the genes responsible for causing A/E lesions including a type III secretion system (T3SS) that encodes the adhesive protein intimin, its receptor named translocated intimin receptor (Tir), and other effector proteins being translocated by the T3SS from the bacterial cytosol into the infected cells [5,6,7,8].

Stxs belong to the group of AB_5_ toxins [9,10] and are released by STEC during colonization into the intestine. According to common assumption, toxin delivery occurs upon bacterial lysis, since no Stx-specific secretion system has been identified yet [1,11]. However, different mechanisms of Stx2 delivery have been described suggesting the involvement of the Stx2-encoding phage induction system and another not further specified Stx2 release system [12]. After translocation across the gut epithelium into the bloodstream, Stx can cause serious extraintestinal complications in the kidney with manifestation of the potentially lethal hemolytic-uremic syndrome (HUS) and, in addition, extrarenal disturbances in the brain including seizures, stroke and coma [13]. Recently, evidence for the involvement of Stx-containing blood cell-derived extracellular vesicles as triggering factors in HUS has been provided that dock preferentially to endothelial cells of target organs [14,15]. Stx-mediated injury of kidney glomerular endothelial cells is the key event for the manifestation of HUS often accompanied by cerebral complications due to damage of the brain endothelium [5,16,17,18,19,20,21,22,23]. Evidence has been provided that renal epithelial cells represent further targets of Stx suggesting contribution of Stx-mediated epithelial cell damage to clinical signs of HUS [22,23,24,25,26,27,28,29]. On the other side, the direct toxic action of Stx at its place of origin in the large intestine, i.e., toxin adhesion to and uptake by colon epithelial cells, is a matter of debate [1].

An early cytotoxicity study indicated Stx-mediated injury of primary cultures of human colonic and ileal epithelial cells, but with the restriction that only 50% of treated cells were affected [30]. Normal human colonic epithelial cells have been reported to lack the glycosphingolipid (GSL) globotriaosylceramide (Gb3Cer), the canonical receptor for the various Stx subtypes [31,32]. On the other hand, the subtypes Stx1(a) and Stx2(a) were shown to bind to colonic epithelial cells in fresh tissue sections, where globotetraosylceramide (Gb4Cer), the less effective Stx receptor GSL, was readily detectable on the cell surfaces of such sections [33]. Moreover, real-time polymerase chain reaction (PCR) analysis revealed expression of Gb3Cer synthase mRNA, suggesting that Gb3Cer may be present in small quantities in normal human colonic epithelia, where it may compete for Stx binding with more abundant Gb4Cer [33]. On the contrary, negative binding experiments of Stx and failure in determining Gb3Cer synthase in normal human colon epithelium have been reported as well [34].

There is no doubt that the human colonic cancer-derived epithelial cell lines Caco-2, HCT-8, HT-29 and T84 investigated so far are sensitive to various Stx subtypes and endowed with Stx GSL receptors suggesting that human enterocytes may be directly damaged by Stx [32,33,35,36,37,38,39], although partly contradictory results were obtained in case of the T84 cell line [31]. Of note, Stx translocation across intact T84 intestinal epithelial cells without apparent cellular disruption [40] and transport of Stx1 via macropinocytosis followed by transcytosis to the basolateral environment have been reported [41], a process that was found to be enhanced in microaerobic environment [42]. Stx translocation across the T84 monolayer and penetration of the toxin seems to occur via a transcellular pathway being independent of the bacterial type III secretion system and A/E lesion formation suggesting that the extent of Stx transcytosis may represent an important indicator of STEC pathogenicity for humans [43].

Lipid rafts are dynamic nanoscale assemblies of sphingolipids (GSLs and sphingomyelin), cholesterol and proteins forming signaling and trafficking platforms in biological membranes [44]. The requirement of lipid rafts has been previously shown for the uptake of Stx1(a) across the apical membrane of Caco-2 cells [45], whereby not only presence of Gb3Cer but also the density of Gb3Cer in lipid rafts may be important for Stx binding as shown for Vero cells [46]. Furthermore, the organization of GSLs including Gb3Cer into lipid rafts might be important to the HUS pathology [47,48]. More precisely, the fatty acid heterogeneity of different Gb3Cer lipoforms may have a functional role regarding the membrane-organizing principle of lipid rafts and perhaps in the pathogenic outcome of HUS [49]. Detergent-resistant membranes (DRMs) are commonly used tools as equivalents of microdomains resembling lipid rafts of the liquid-ordered membrane phase [50,51] and have been successfully applied in the analysis of Stx-receptor interactions and retrograde trafficking of the toxin [46,52]. 

Cattle are a major reservoir of human-pathogenic STEC strains, which can persistently colonize the gut representing a major source of human infections [53]. Colonization of cattle occurs predominantly in the large intestine, where the pathogens may especially target epithelial cells in the terminal rectum [54]. STEC strains that cause hemorrhagic colitis and HUS in humans express high levels of Stx and cause A/E lesions in intestinal epithelial cells [55]. Cattle were considered for a long time resistant to the detrimental effects of Stx, although substantial evidence has been provided in the past that different types of Stx-targeted cells do exist in cattle, including peripheral and intestinal lymphocytes, certain colonic epithelial cells and macrophage-like cells [53,56,57,58,59]. Importantly, the presence of the Stx-receptor Gb3Cer has been detected in the bovine small and large intestine as well as discrete cell subsets in the bovine kidney and submucosal lymphoid cells, but not in the vasculature [60]. Thus, the presumed general absence of Stx receptor GSLs on bovine vascular endothelium could contribute to the apparent resistance of cattle to systemic effects of Stx. This suggestion is supported by an early study, in which the presence of only trace amounts of Gb3Cer and lack of elongated Stx-binding globo-series GSLs has been demonstrated for bovine aortic endothelial cells, which contain mostly neolacto-series GSLs as the typical GSLs [61]. This fact explains the low susceptibility of Stx2e towards bovine aortic endothelial cells [62] because neolacto-series GSLs exhibit considerably different sugar epitopes when compared to globo-series GSLs [63,64,65,66], and this excludes them definitively as Stx-binding GSLs. The distinct cellular repertoire of GSLs might be the reason that Stx-targeted cells and resulting Stx-mediated effects in cattle differ from those implicated in human disease [53].

Since receptor GSLs of Stx of human colon epithelial cells have neither been identified nor structurally characterized until today, the aim of this study was the identification and structural characterization of Stx-binding GSLs of primary human colon epithelial cells (pHCoEpiCs) grown under serum-free conditions as well as their localization in membrane microdomains using DRMs and nonDRMs as equivalents for the liquid-ordered and liquid-disordered membrane phase, respectively. Thin-layer chromatography (TLC) overlay immunodetection with anti-Gb3Cer and anti-Gb4Cer antibodies as well as overlay detection using the Stx subtypes Stx1a, Stx2a and Stx2e were employed together with sophisticated mass spectrometry techniques for GSL analysis. Identified receptors were Gb3Cer and Gb4Cer occurring as lipoforms having fatty acids that varied in chain length from C16 up to C24 carbon atoms and an invariable sphingosine (d18:1) portion in the ceramide (Cer) lipid core. This is the first report that describes existence and structures of Stx-binding Gb3Cer and Gb4Cer species and their prevalence in DRMs prepared from pHCoEpiCs as well as their cellular sensitivity towards affinity-purified Stx1a, Stx2a and Stx2e subtypes. 

## 2. Results

The first batch of cell cultures was produced with pHCoEpiCs grown under serum-free conditions. Since it is known that serum contains GSLs and because such exogenous GSLs can be incorporated by in vitro propagated cells, this approach was initially undertaken. We isolated Stx-binding GSLs from pHCoEpiCs cultivated without serum and performed full structural characterization of their structures. The isolated GSLs represent the genuine Stx-receptors of human colon epithelium, which have never been scrutinized before in such detail as shown in this study. However, serum deprivation results in slower growth of the cells and a limited amount of cell mass for extensive investigations. Thus, some investigations were performed using lipid material from pHCoEpiCs grown with low concentrations (2.5%) of fetal bovine serum (FBS) as mentioned in the following sections. Cells of early passage 5 and later passages 10 and 14 are shown in Figure S1 of the Supplementary Materials indicating the phenotypical/morphological changes of pHCoEpiCs occurring during prolonged cultivation. Analytical and cytological experiments were performed with pHCoEpiCs at early passages, i.e., between passage 4 and passage 5. 

### 2.1. Detection of Stx-Binding GSLs in pHCoEpiCs by Means of Immunochemical Analysis

**Figure 1:**
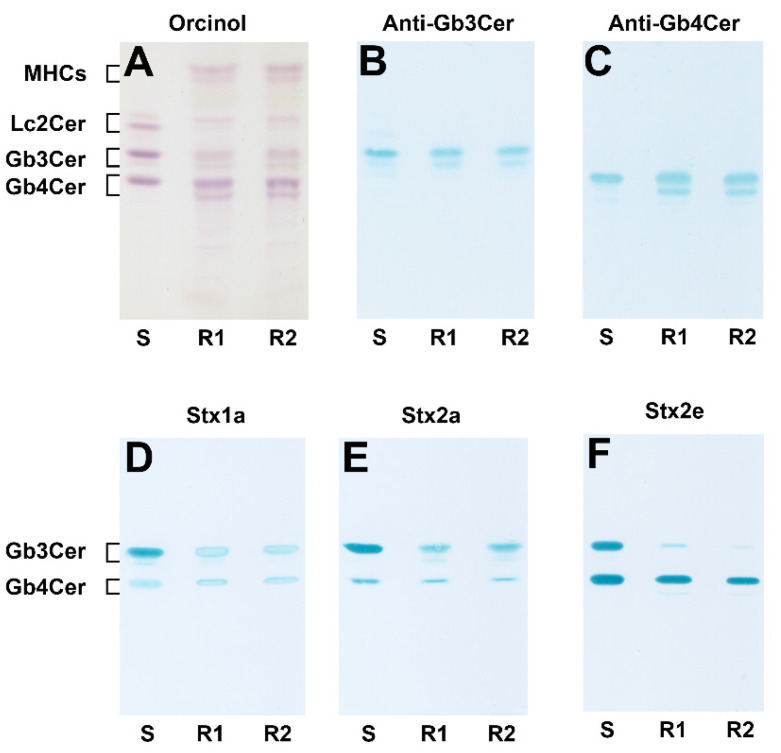
Orcinol stain (**A**) and antibody- (**B**,**C**) and Stx-mediated TLC overlay detection (**D**–**F**) of Gb3Cer and Gb4Cer in GSL preparations of serum-free cultivated pHCoEpiCs. The applied GSL quantities correspond to 5 × 10^6^ cells for the orcinol stain (**A**), 5 × 10^5^ cells for the anti-Gb3Cer (**B**) and anti-Gb4Cer overlay assay (**C**), respectively, and to 1 × 10^6^ cells for the Stx overlay assays (**D**–**F**). Deployed amounts of the standard mixture (S): 6 µg (**A**), 2.5 µg and 0.25 µg (**B** and **C**, respectively), and 2.6 µg (**D**–**F**) of a neutral GSL preparation containing equimolar amounts of Gb3Cer and Gb4Cer.

Figure 1 shows the orcinol stain of the neutral GSLs from serum-free cultivated pHCoEpiCs and the immunochemical identification of Gb3Cer and Gb4Cer performed in parallel with specific antibodies as well as their recognition by the three employed Stx subtypes Stx1a, Stx2a and Stx2e. The neutral GSLs from two independently produced biological replicates (R1 and R2) were isolated from the lipid extracts and purified by anion-exchange chromatography. The orcinol-stained TLC-separated GSLs of pHCoEpiCs (Figure 1A) suggest the presence of an abundant Gb4Cer and a comparably weak Gb3Cer double band, respectively. The supposed glycan epitopes could be confirmed using the anti-Gb3Cer (Figure 1B) and the anti-Gb4Cer antibody (Figure 1C), indicating clearly immunostained GSL doublets. Experience from previous GSL analyses of various types of human cells suggests ceramide lipoforms harboring a long-chain C24 fatty acid in the upper and a short-chain C16 fatty acid in the lower band of the respective Gb3Cer and Gb4Cer double bands with a uniform sphingosine (d18:1) as the sphingoid base. Stx1a and Stx2a exhibit preferential recognition of Gb3Cer, especially of the lipoform with the long-chain fatty acid in the upper band, accompanied by weak but clearly detectable binding to the Gb4Cer upper band as shown in Figure 1D and Figure 1E, respectively. In contrast, Stx2e is characterized by preferred binding towards Gb4Cer and extremely low interaction with Gb3Cer (Figure 1F). Threshold concentrations of the minor Gb3Cer and Gb4Cer species carrying the short-chain fatty acid appearing as lower GSL bands of the doublets obviously prevented binding of the three Stx-subtypes.

### 2.2. Mass Spectrometric Determination of Stx Receptors Gb3Cer and Gb4Cer of pHCoEpiCs

**Figure 2:**
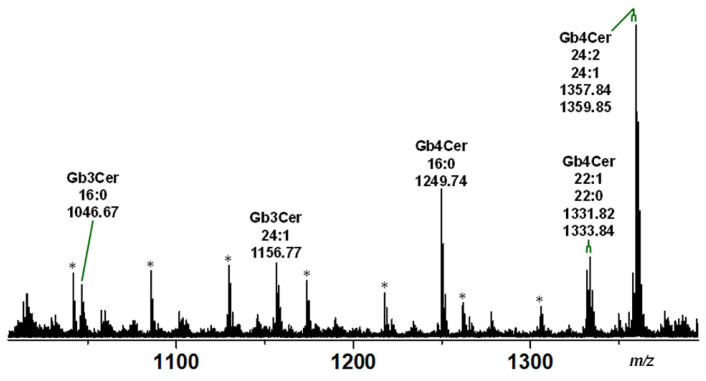
Overview MS^1^ spectrum of Stx receptor GSLs Gb3Cer and Gb4Cer of serum-free cultivated pHCoEpiCs. The spectrum was obtained from a GSL preparation of replicate 2 (R2; see Figure 1) showing the various lipoforms of Gb3Cer and Gb4Cer harboring sphingosine (d18:1) as the sole sphingoid base and variable fatty acids as indicated. All GSLs were detected as monosodiated species using the positive ion mode and are listed in Table 1. The [M+Na]^+^ ions of Gb3Cer at *m/z* 1046.67 and 1156.77 and those of Gb4Cer at *m/z* 1249.74 and 1357.84/1359.85 were selected for MS^2^ analysis as shown in Figure 3 and Figure 4, respectively. The asterisks mark polyethylene glycols (PEGs) appearing as serial contaminations in the GSL preparation.

The structural characterization of the proposed Gb3Cer and Gb4Cer species by means of high-resolution electrospray ionization mass spectrometry (ESI-MS) employing MS^1^ and MS^2^ analysis is provided in the following. Two independent biological replicates of serum-free grown pHCoEpiCs were applied and representative data of replicate 2 are shown. Identified Gb3Cer lipoforms were Gb3Cer (d18:1, C16:0) and Gb3Cer (d18:1, C24:1) and the Gb4Cer lipoforms Gb4Cer (d18:1, C16:0), Gb4Cer (d18:1, C22:1/C22:0) and Gb4Cer (d18:1, C24:2/C24:1) detected as sodium adducts [M+Na]^+^ as shown in the spectrum presented in Figure 2 (for listing see Table 1). The MS^1^ spectrum indicated high abundance of Gb4Cer species and lower abundance of the Gb3Cer variants in compliance with the estimated relative content deduced from the orcinol stain of the TLC-separated GSLs (see Figure 1A). The counterpart MS^1^ spectrum of replicate 1 is provided as Figure S2 in the Supplementary Materials, showing the same Gb3Cer and Gb4Cer lipoforms and thus great consistency of the two replicates. Importantly, the ceramides of Gb3Cer and Gb4Cer with saturated C24:0 fatty acid were not detectable in the GSL isolates of both replicates, most likely due to extremely low abundance in the GSL preparation of total cells.

**Figure 3:**
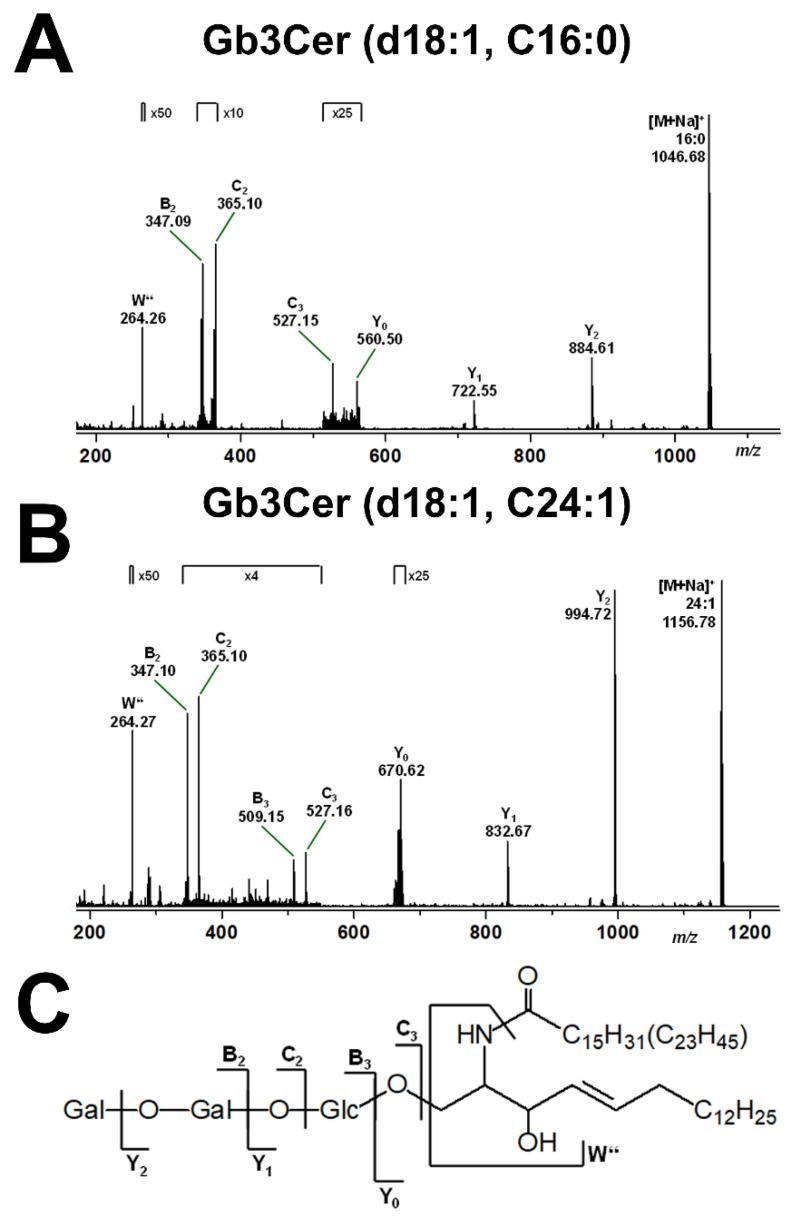
MS^2^ spectrum of selected Gb3Cer (d18:1, C16:0) (**A**) and Gb3Cer (d18:1, C24:1) (**B**) obtained from serum-free cultivated pHCoEpiCs and the explanatory fragmentation scheme (**C**). The MS^2^ spectra were obtained from a GSL preparation of replicate 1 (R1; see Figure 1) using the positive ion mode. [M+Na]^+^ adducts of the various Gb3Cer lipoforms harboring sphingosine (d18:1) and variable fatty acids as indicated were fragmented by means of collision-induced dissociation (CID).

Mass spectrometric fragmentation analyses are exemplarily shown for the Gb3Cer lipoforms Gb3Cer (d18:1, C16:0) and Gb3Cer (d18:1, C24:1) in Figure 3A and Figure 3B, respectively, with an explanatory fragmentation scheme in Figure 3C regarding the fragment ions obtained by collision-induced dissociation (CID) experiments. This fragmentation technology allows for individual GSL analysis with respect to monosaccharide sequencing of the oligosaccharide portion and determining the ceramide structure composed of a sphingoid base and a fatty acid moiety. Since the pHCoEpiCs were propagated in serum-free medium, the uptake of exogenous GSLs from serum supplement can be excluded and identified Stx receptor GSLs can be considered as true GSLs of colon epithelial cells. 

**Figure 4:**
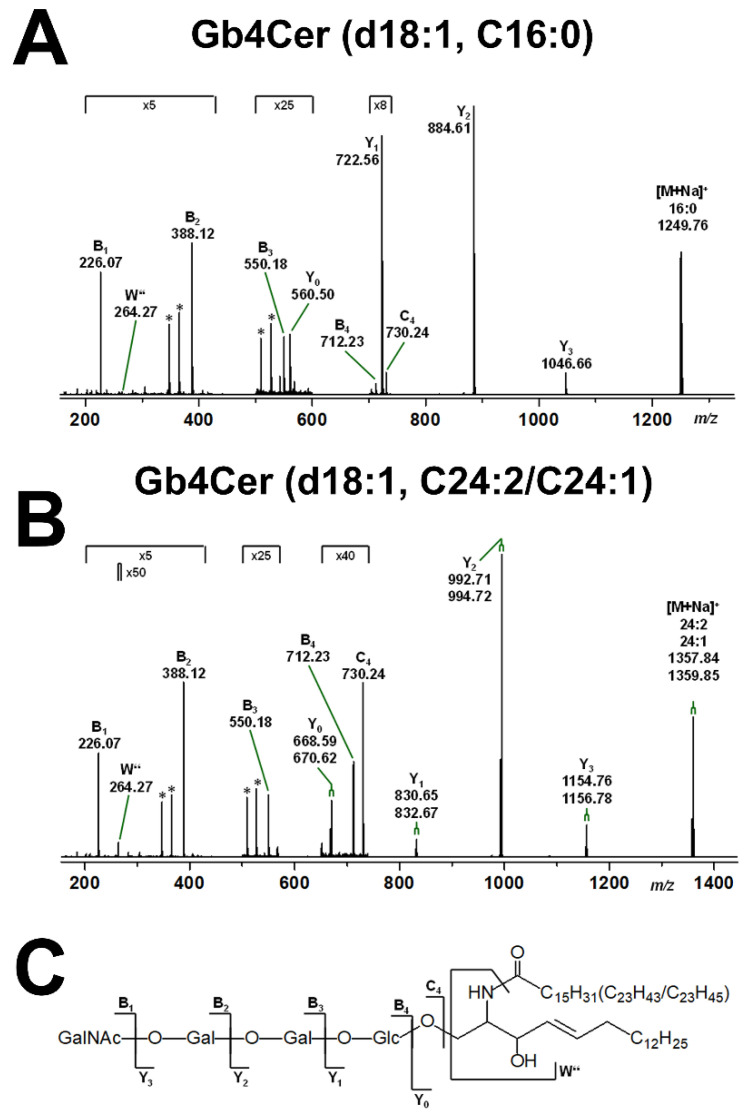
MS^2^ spectrum of selected Gb4Cer (d18:1, C16:0) (**A**) and Gb4Cer (d18:1, C24:2/C24:1) (**B**) obtained from serum-free cultivated pHCoEpiCs and the explanatory fragmentation scheme (**C**). The MS^2^ spectra were obtained from a GSL preparation of replicate 1 (R1; see Figure 1) using the positive ion mode. [M+Na]^+^ adducts of the various Gb4Cer lipoforms harboring sphingosine (d18:1) and variable fatty acids as indicated were fragmented by means of collision-induced dissociation (CID). The asterisks mark internal fragments.

Parallel CID experiments were performed with the Gb4Cer lipoforms and examples of MS^2^ spectra are shown for the Gb4Cer lipoforms Gb4Cer (d18:1, C16:0) and Gb4Cer (d18:1, C24:2/C24:1) in Figure 4A and Figure 4B, respectively, accompanied by the supporting fragmentation scheme depicted in Figure 4C. Again, cultivation of the pHCoEpiCs in serum-free medium proved the various Gb4Cer species as inherent GSLs of colon epithelial cells.

### 2.3. Detection of Gb3Cer, Gb4Cer, and Cholesterol in Detergent-Resistant Membranes (DRMs) and nonDRMs Prepared from pHCoEpiCs

**Figure 5:**
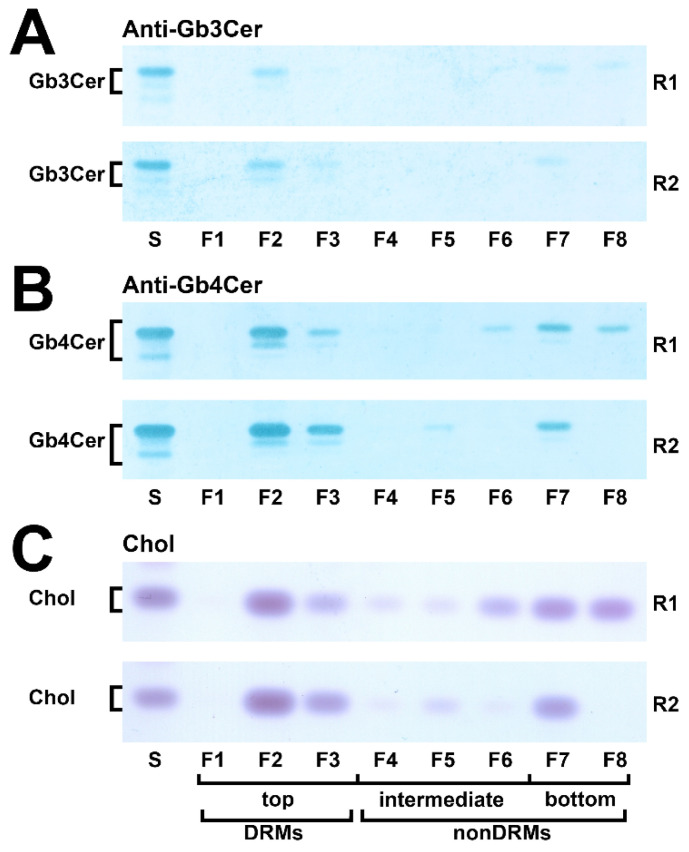
Distribution of Gb3Cer (**A**), Gb4Cer (**B**) and cholesterol (**C**) among sucrose gradient fractions F1 to F8 obtained from pHCoEpiCs. Anti-Gb3Cer (**A**) and anti-Gb4Cer (**B**) antibodies were used for immunochemical detection and manganese(II)chloride for cholesterol (Chol) detection in samples of two independent biological replicates (R1 and R2) obtained from pHCoEpiCs cultivated in medium with low serum. Each gradient preparation corresponds to 5 × 10^6^ cells. Standard (S) equivalents of 2 µg and 0.2 µg of reference neutral GSLs from human erythrocytes and 1 µg of reference cholesterol (Chol) were employed as positive controls for the anti-Gb3Cer and anti-Gb4Cer TLC overlay assay and the cholesterol detection, respectively. DRMs, detergent resistant membranes.

Due to the relatively low recovery of cellular mass from slowly growing cells in serum-free culture medium, two replicates of pHCoEpiCs were produced in medium supplemented with low concentration of serum, i.e., 2.5% fetal bovine serum (termed as “low-serum conditions”), in order to produce sufficient cellular material for the preparative isolation of DRM and nonDRM fractions from sucrose gradients. The distribution of Gb3Cer, Gb4Cer, and cholesterol within the DRM F1-F3 (top), nonDRM F4-F6 (intermediate) and nonDRM F7-F8 (bottom) fractions of replicate 1 (R1) and replicate 2 (R2) of pHCoEpiCs is shown in Figure 5. 

**Figure 6:**
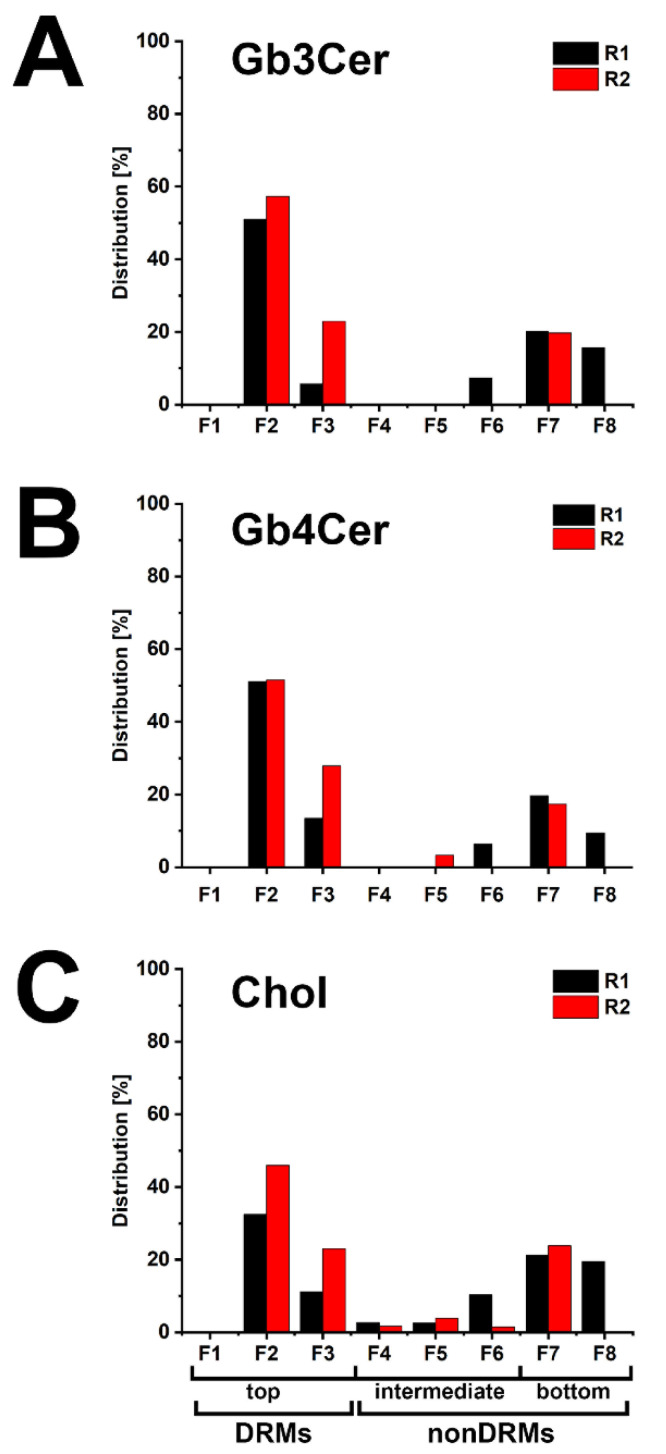
Portrayal of the distribution of Gb3Cer (**A**), Gb4Cer (**B**) and cholesterol (**C**) among sucrose gradient fractions F1 to F8 obtained from pHCoEpiCs. The anti-Gb3Cer and anti-Gb4Cer detected immunopositive GSL bands and the manganese(II)chloride-stained cholesterol bands shown in Figure 5 were scanned and normalized to 100% for each distribution pattern of Gb3Cer, Gb4Cer and cholesterol compiled for the two independent biological replicates (R1 and R2). A list of measured values is provided in Table S1 of the Supplementary Materials.

The distribution pattern of Gb3Cer in both replicates indicated preference of Gb3Cer in DRM fraction F2 and low amounts in the nonDRM fractions F7 and F8 (Figure 5A). Similarly, strong immunopositive Gb4Cer bands were detected in DRMs F2 and F3 and lower content in nonDRMs F7 and F8 (Figure 5B), indicating preferred occurrence of Gb4Cer in DRMs. Although not quantitatively determined, moderate and strong positive antibody reactions with Gb3Cer and Gb4Cer, respectively, correlates with the orcinol stain of these two GSLs in total GSLs from pHCoEpiCs (see Figure 1A). The data of relative densitometric quantification are depicted as bar diagram in Figure 6 and the exact measurement values are listed in Table S1 in the Supplementary Materials.

The averaged values of Gb3Cer of the summed top fractions (DRMs F1-F3) of replicate 1 and replicate 2 account for 68.5% and those of the summed bottom fractions (nonDRMs F7-F8) of the two replicates amount to 27.9% (for calculation refer to Table S1 of the Supplementary Materials). Similar partition values were calculated from densitometric scanning of Gb4Cer with an average of 71.9% in the added top fractions (DRMs F1–F3) and averaged 23.3% in the added bottom fractions (nonDRMs F7-F8) when compared to the distribution pattern of Gb3Cer. Cholesterol disseminates on average to 56.4% to the summed top fractions (DRMs F1-F3) and to averaged 32.2% to the summed bottom fractions (nonDRMs F7-F8). These values suggest association of Gb3Cer and Gb4Cer with microdomains in the liquid-ordered membrane phase, substantiated by peak prevalence of averaged 54.2% of Gb3Cer, 51.3% of Gb4Cer and 39.3% of cholesterol in the single classical DRM fraction F2 of the eight gradient fractions (calculated from relative scanning values listed in Table S1 of the Supplementary Materials). Collectively, prevalence of Gb3Cer and Gb4Cer in the DRMs suggest their possible association with membrane clusters also known as lipid rafts.

### 2.4. Gb3Cer and Gb4Cer Lipoforms Derived from Detergent-Resistant Membranes (DRMs) of pHCoEpiCs Grown under Low-Serum and Serum-Free Conditions

Since serum supplementation of the growth medium may cause different GSL patterns due to possible uptake of exogenous GSLs from serum, the exact structures of the Gb3Cer and Gb4Cer lipoforms derived from the DRM F2 fractions of pHCoEpiCs propagated with sparse serum and without serum were scrutinized by MS^1^ and MS^2^ analysis. 

**Figure 7:**
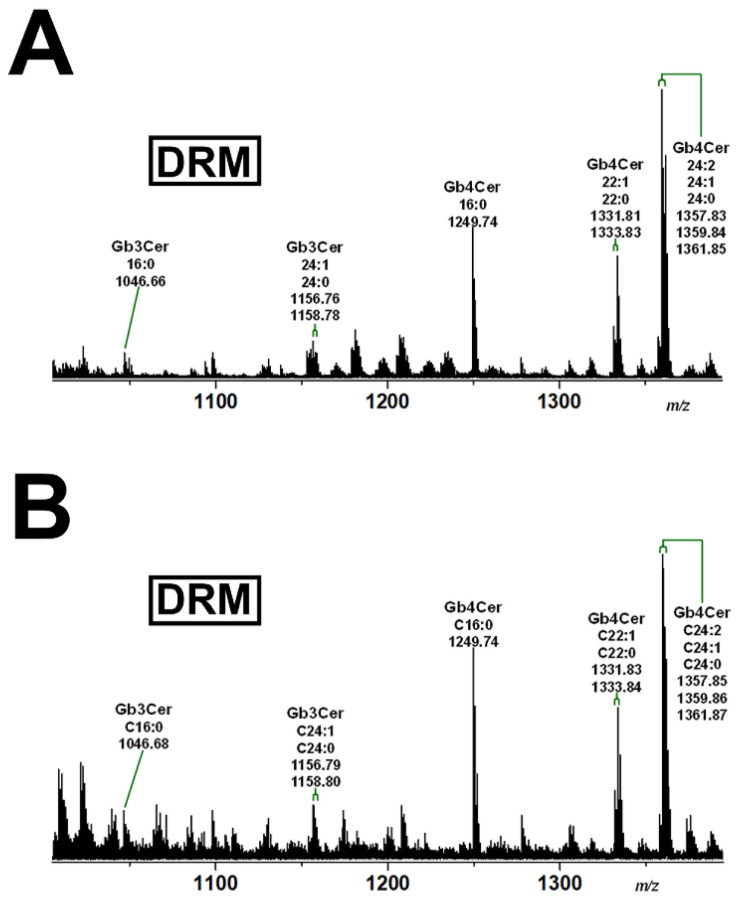
Overview mass spectra of the Gb3Cer and Gb4Cer lipoforms obtained from the DRM F2 fractions of pHCoEpiCs propagated in low-serum (**A**) and serum-free medium (**B**). The extended spectra of Gb3Cer and Gb4Cer in the GSL preparation of DRM fraction F2 in comparison to total GSLs derived from serum-free cultivated cells are depicted in Figure 8.

**Figure 8:**
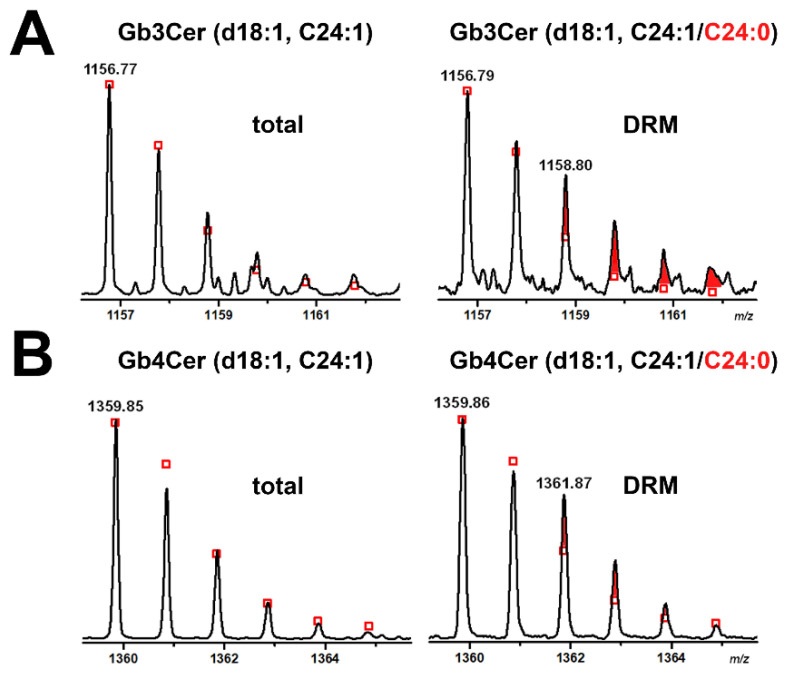
Expanded molecular ion region of Gb3Cer C24 species detected in the total GSL preparation and the GSL sample of DRM fraction F2 (**A**) and the Gb4Cer counterparts (**B**) of serum-free cultivated pHCoEpiCs. The calculated isotope distribution of Gb3Cer and Gb4Cer with Cer (d18:1, C24:1) of the total GSL preparation (left side of panels A and B, respectively) and Gb3Cer and Gb4Cer with Cer (d18:1, C24:1/C24:0) of the GSL sample of DRM fraction F2 (right side of panels A and B, respectively) is marked with open red squares. The red coloured areas at the tips of the signal peaks indicate the additionally occurring Gb3Cer and Gb4Cer lipoforms with Cer (d18:1, C24:0) moieties.

Figure 7 shows the MS^1^ spectrum of Gb3Cer and Gb4Cer obtained from DRM fraction F2 of low serum-grown cells (Figure 7A) and the MS^1^ spectrum of Gb3Cer and Gb4Cer of DRM fraction F2 of serum-free propagated cells (Figure 7B) both derived from replicate 2. The pattern of both approaches was identical showing the same Gb3Cer and Gb4Cer lipoforms, thus excluding an influence of low-serum supplementation on the expression of the Stx receptor GSLs when compared to serum-free conditions. Importantly, both spectra revealed the presence of Gb3Cer and Gb4Cer variants with saturated C24:0 fatty acid, which were not detectable in total GSLs of pHCoEpiCs (see Figure 2). To illustrate this, the expanded molecular ion regions of Gb3Cer and Gb4Cer C24-species derived from total GSLs (see Figure 2) and those obtained from DRM F2 fraction (see Figure 7B), in both cases received from serum-free grown pHCoEpiCs, are shown in Figure 8. The extended Gb3Cer spectrum from total GSLs revealed exclusive presence of Gb3Cer (d18:1, C24:1) indicated by the monoisotopic peak at *m/z* 1156.77, whereas the calculated isotope pattern (depicted as red squares) of the DRM counterpart indicated occurrence of Gb3Cer (d18:1, C24:0) at *m/z* 1158.80 in addition to Gb3Cer (d18:1, C24:1) at *m/z* 1156.80 as demonstrated in Figure 8A. For reasons of clarification, the newly appearing Gb3Cer lipoform with Cer (d18:1, C24:0) in DRM fraction F2 is marked as a triplet of three red coloured tips of the signal peaks in the extended spectrum. The same feature holds true for the Gb4Cer lipoforms detected in the DRM fraction of pHCoEpiCs as shown in Figure 8B. However, in total GSLs, the isotope pattern of Gb4Cer (d18:1, C24:1) with monoisotopic peak at *m/z* 1359.85 was in excellent agreement with the calculated one and did not exhibit the variant with C24:0 fatty acid. The Gb4Cer (d18:1, C24:0) species appearing as monoisotopic peak at *m/z* 1361.87 in addition to Gb4Cer (d18:1, C24:1) at *m/z* 1359.86 is marked as a triplet of three red coloured tips of the peak areas being characteristic for the DRM F2 fraction. Collectively, enrichment of GSLs with Cer (d18:1, C24:0) in the DRM F2 fractions being not detectable in the total GSL preparation were found as unique lipoforms in the DRM F2 fractions suggesting these lipoforms of Gb3Cer and Gb4Cer as possible markers for lipid rafts of pHCoEpiCs. 

### 2.5. Identification of DRM and nonDRM Phospholipids

**Figure 9:**
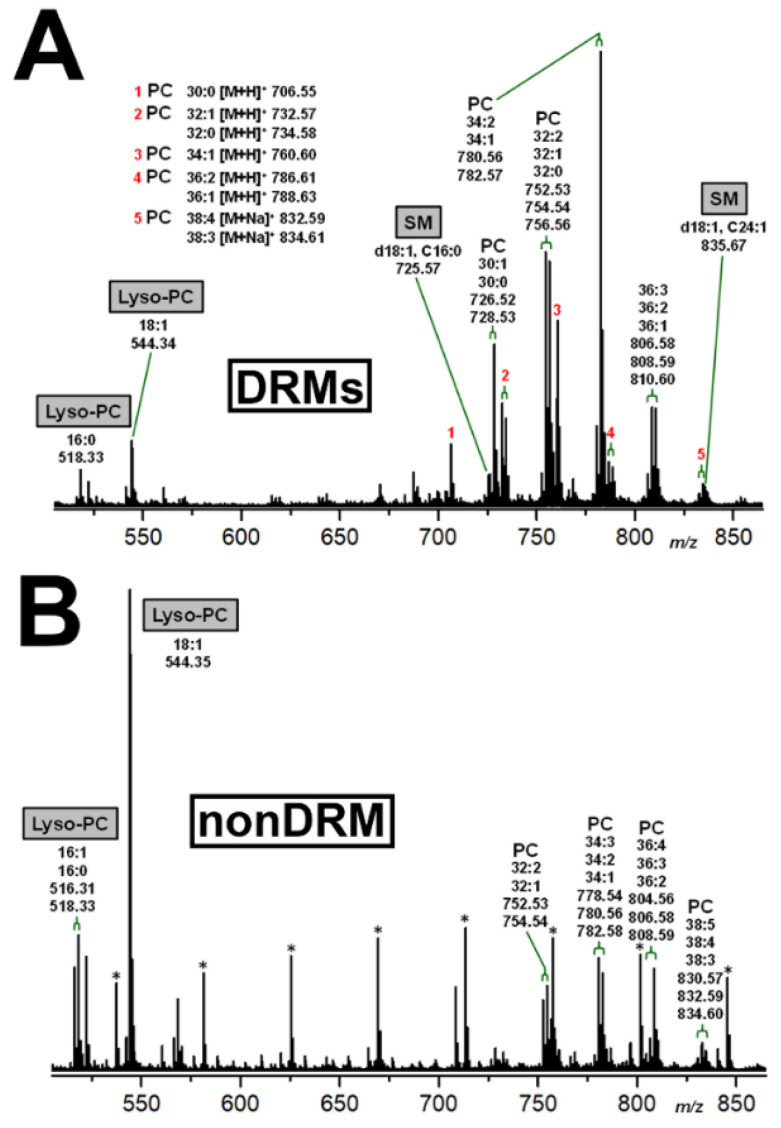
Phospholipid MS^1^ spectra of DRM (**A**) and nonDRM fractions (**B**) derived from low-serum cultivated pHCoEpiCs. MS spectra of combined lipid extracts from DRM F2 and F3 and nonDRM F7 fractions of replicate 1 obtained in the positive ion mode are exemplarily shown. Unless otherwise stated, the detected sodiated species ([M+Na]^+^) are assigned. The SM species occurring only in DRMs are considered as markers of the liquid-ordered membrane phase and highlighted as grey boxes. The lyso-PCs in the nonDRM fraction can be considered as markers for the liquid-disordered membrane phase due to their exorbitant content in the nonDRM fraction F7 and are framed in grey boxes as well. The asterisks mark polyethylene glycols (PEGs) appearing as serial contaminations in the lipid preparation.

Lipid extracts of DRM and nonDRM fractions obtained from pHCoEpiCs were submitted to ESI-MS analysis using the positive ion mode aimed at identification of phospholipid markers that might be possibly associated with lipid rafts. MS^1^ spectra of combined lipid extracts from DRM F2 and F3 and nonDRM F7 fractions derived from low-serum cultivated pHCoEpiCs of replicate 1 are exemplarily depicted in Figure 9, showing the distinctive phospholipids of DRMs (Figure 9A) and those of the nonDRM fraction (Figure 9B). Various phosphatidylcholine (PC) lipoforms dominated in the lipid preparation of DRMs (Figure 9A), videlicet those with a 34:2/34:1 fatty acid doublet and a 32:2/32:1/32:0 fatty acid triplet at *m/z* 780.56/782.57 and 752.53/754.54/756.56, respectively. These predominant PL species were accompanied by less abundant PC species harboring 30:1/30:0 and 36:3/36:2/36:1 lipid anchors detected at *m/z* 726.52/728.53 and 806.58/808.58/810.60, respectively. Minor species were lyso-PC (16:0) and lyso-PC (18:1) identified by *m/z* 518.33 and 544.34, respectively. Sphingomyelins (SMs) with Cer (d18:1, C24:1) and Cer (d18:1, C16:0) at *m/z* 835.67 and 725.57 can be considered as marker PLs of the DRMs. On the other hand, lyso-PC (18:1) and lyso-PC (16:1, 16:0) with *m/z* signals of 544.35 and 516.31/518.38, respectively, were the major PLs in the nonDRM fraction F7 (Figure 9B), accompanied by similar but less abundant PC lipoforms found in the DRMs, but appearing here in much lower concentrations. Importantly, SMs were absent in the nonDRM fraction. The high reproducibility is demonstrated by almost identical PL pattern obtained for replicate 2 of pHCoEpiCs provided in Figure S3 of the Supplementary Materials.

### 2.6. Stx-Mediated Cellular Damage of pHCoEpiCs

**Figure 10:**
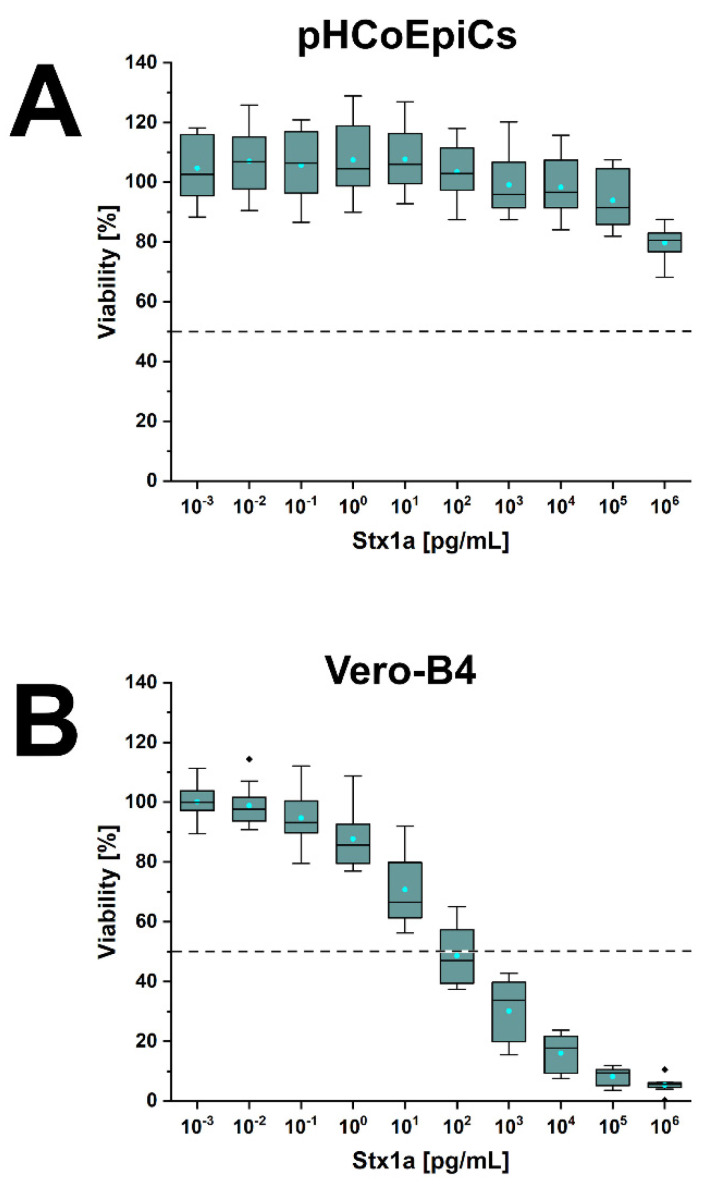
Cytotoxic effect of Stx1a towards pHCoEpiCs (**A**) and Vero-B4 reference cells (**B**). The cell-damaging activity was analyzed with the crystal violet assay, and absorption readouts of Stx1a-challenged cells are presented as box plots. Percentages of cell damage were determined in relation to parallel cell cultures without toxin serving as 100% viability control.

**Figure 11:**
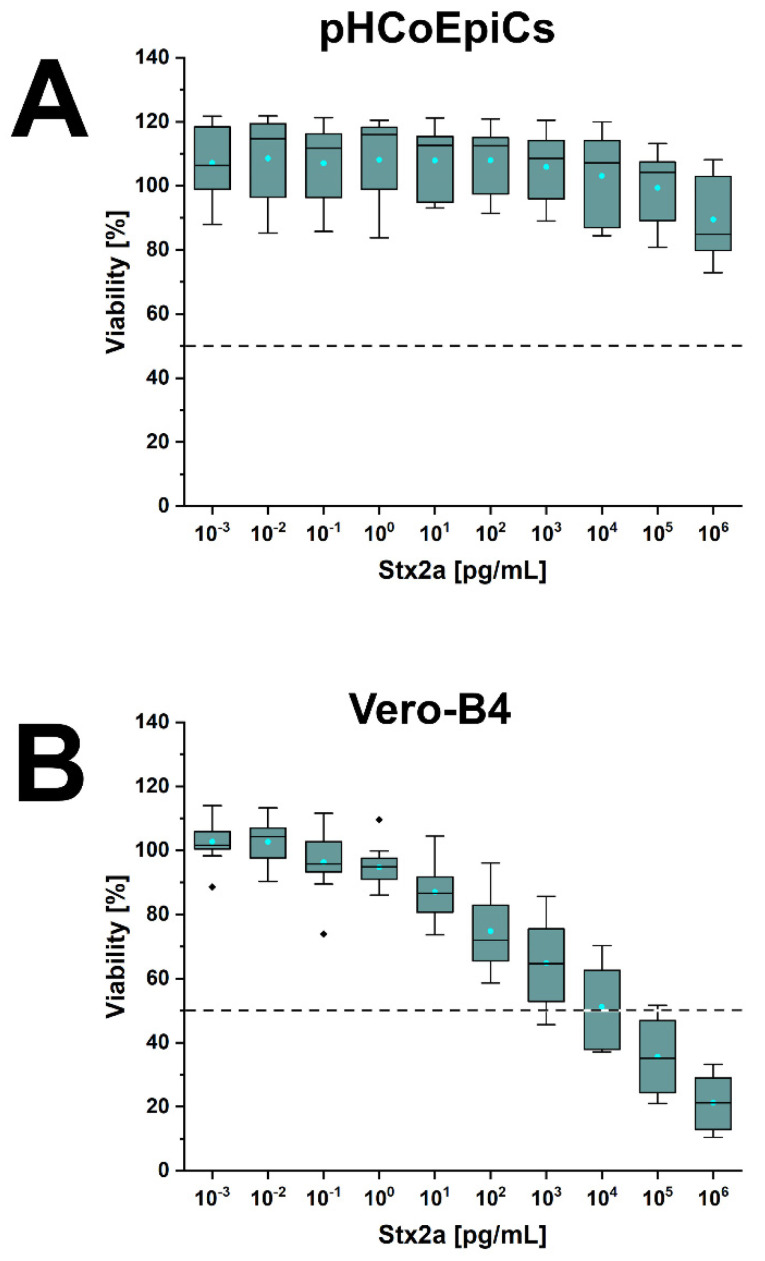
Cytotoxic effect of Stx2a towards pHCoEpiCs (**A**) and Vero-B4 reference cells (**B**). The cell-damaging activity was analyzed with the crystal violet assay, and absorption readouts of Stx2a-challenged cells are presented as box plots. Percentages of cell damage were determined in relation to parallel cell cultures without toxin serving as 100% viability control.

**Figure 12:**
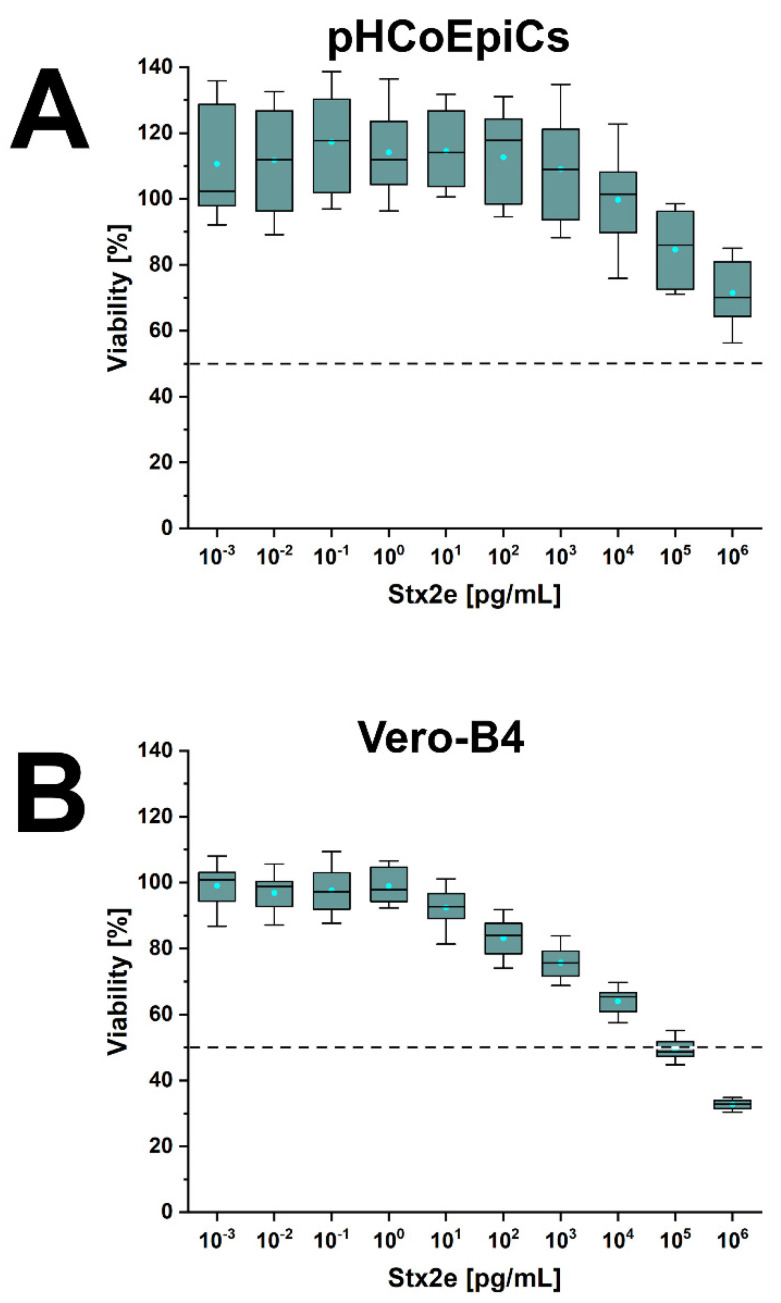
Cytotoxic effect of Stx2e towards pHCoEpiCs (**A**) and Vero-B4 reference cells (**B**). The cell-damaging activity was analyzed with the crystal violet assay, and absorption readouts of Stx2e-challenged cells are presented as box plots. Percentages of cell damage were determined in relation to parallel cell cultures without toxin serving as 100% viability control.

Cells of early passages (4th and 5th passage) were exposed to increasing concentrations of Stx1a, Stx2a and Stx2e. The lowest applied toxin concentration was 10^−3^ pg/mL (appropriate to 1 fg/mL) and the highest concentration applied was 10^6^ pg/mL (corresponding to 1 µg/mL). The benchmark of 100% viability were untreated cells, and survival of Stx-treated pHCoEpiCs was determined related to these control cells. The survival rate of pHCoEpiCs exposed to the serially increased concentrations of Stx1a is shown in Figure 10. The course of Stx1a treatment indicates a high degree of toxin tolerance with a minimal cell damage response in a concentration range between 10^3^ and 10^5^ pg/mL, which ended with decreased viability of 79.7 ± 5.1% upon application of the highest toxin concentration of 1 µg/mL (Figure 10A). Reference Vero-B4 cells exhibited strong sensitivity towards Stx1a, exhibiting a 50% cytotoxic dose (CD_50_) of 9.4 × 10^1^ pg/mL and a final cell survival of 5.4 ± 2.3% (Figure 10B). A similarly low sensitivity of the pHCoEpiCs was observed for Stx2a with a cell survival rate of 89.5 ± 12.4% after exposure to the uppermost toxin concentration applied (Figure 11A). The CD_50_ value of Stx2a for Vero-B4 cells was 1.8 × 10^4^ pg/mL indicating a rather moderate sensitivity of the reference cell line towards Stx2a corresponding to a final viability of 21.2 ± 9.0% (Figure 11B). Interestingly, the only significant cytotoxic effect towards pHCoEpiCs was determined for Stx2e with cell survival rates of 84.6 ± 11.3% and 71.5 ± 10.4% received upon treatment with 10^5^ and 10^6^ pg/mL, respectively (Figure 12A). Vero-B4 cells exhibited a rather low sensitivity against Stx2e with a CD_50_ value amounting to 9.6 × 10^4^ pg/mL and a final cell survival of 32.7 ± 1.5% (Figure 12B).

In summary, pHCoEpiCs were largely tolerant against the cytotoxic action of Stx1a and Stx2a, showing only a mild but significant cytotoxic response towards Stx2e. However, using 1 µg/mL as the highest toxin concentrations, no 50% cytotoxic doses could be determined.

## 3. Discussion

In contrast to numerous previous studies employing the colon tumor-derived epithelial cell lines Caco-2, HCT-8, HT-29 and T84, we used primary human colon epithelial cells (pHCoEpiCs) because primary cells in general preserve much better the genetic signature of healthy cells of a donor than tumor descendants. However, previous studies were helpful because they indicated the principal capability of colon cancer cell lines to produce the globo-series GSLs Gb3Cer and Gb4Cer [31,32,33,35,36,37,38,39], although their cellular endowment with Stx receptors might differ from that of primary colonic epithelial cells. In this study, we used primary cells, which were propagated for the identification and precise structural characterization of the various Gb3Cer and Gb4Cer lipoforms without serum. The omission of serum results in slowed cell growth but excludes possible cellular uptake and membrane incorporation of exogenous GSLs from supplemental serum commonly used in concentrations between 5% to 10% for in vitro cell cultivation. The serum-free propagated pHCoEpiCs were found to contain Gb4Cer as the prevalent neutral GSL carrying ceramides with uniform sphingosine (d18:1) and variable C16:0, C22:1/C22:0 or C24:2/C24:1 fatty acyl chains. The renowned Stx1(a)/Stx2(a) receptor GSL Gb3Cer was detected as less abundant GSL with unchanged sphingosine (d18:1) and variable C16:0 or C24:1 fatty acid. Gb3Cer and Gb4Cer lipoforms with C24:0 fatty acid were detected as minor compounds only in DRMs, obviously enriched in this type of microdomains, but being undetectable in the total GSL preparation of the cells. The identified Gb3Cer and Gb4Cer lipoforms in the total GSL preparation of pHCoEpiCs were identical to those previously detected in primary human renal cortical epithelial cells (pHRCEpiCs) [28] and primary human proximal tubular epithelial cells (pHPTEpiCs) [29]. However, pHCoEpiCs differed from kidney epithelial cells by the lack of globopentaosylceramide (Gb5Cer, Galβ1-3GalNAcβ1-3Galα1-4Galβ1-4Glcβ1-1Cer), which was found in both kidney epithelial cells as minor constituent and is known as an additional receptor (besides Gb3Cer and Gb4Cer) for promiscuous Stx2e [67]. 

For a long time, the presence of Gb3Cer and/or Gb4Cer in human colon epithelial cells was obscure due to partly controversial results obtained by different research groups. The previous finding that normal human colonic epithelial cells may lack Gb3Cer, the renowned receptor for the various Stx subtypes [31,32], can be explained by the very low content of this GSL species hardly detectable in common cell- or GSL-preparations. On the other hand, expression of Gb3Cer synthase mRNA was detected by means of real-time PCR analysis, suggesting the existence of Gb3Cer, at least in small amounts, in normal human colonic epithelium along with dominating Gb4Cer [33]. Our optimized combined procedure of sensitive TLC immunodetection and sophisticated mass spectrometric analysis allowed the identification and precise structural characterization of low abundant but highly effective Stx-binding Gb3Cer and of high abundant but less effective Stx-binding Gb4Cer. Importantly, Gb4Cer has been previously detected on the surface of human colonic tissue sections [33], a preliminary result that could be now confirmed by us for pHCoEpiCs with precise structural data. However, supposed heterogeneity of the GSL profiles and possible differing repertoires of GSL synthases of individual donors cannot be excluded and gives the explanation of failure in detection of Gb3Cer and the corresponding Gb3Cer synthase in normal human colon epithelium that has been also reported [34]. Consequently, future research projects should encompass a larger batch of colon epithelial cells derived from donors of different age and gender that should clarify such at this time open questions.

It is generally known that Stx1a and Stx2a subtypes are associated with severe EHEC infections in humans [68], whereas Stx2e is the key virulence factor of STEC causing porcine edema disease [69], and its detection is associated with mild disease in humans [70]. Unexpectedly, the swine-pathogenic Stx2e-subtype exhibited notable toxicity towards pHCoEpiCs, which, on the other hand, were largely robust against the human-pathogenic Stx1a- and Stx2a-subtype. However, the dominance of Gb4Cer might be the reason for this behaviour because Gb4Cer represents the preferred receptor of Stx2e when compared to Gb3Cer [71,72]. Thus, Stx2e exhibited a stronger cell-damaging effect than Stx1a and Stx2a, most likely based on the much higher content of Gb4Cer versus Gb3Cer in pHCoEpiCs. The relatively high content of Gb4Cer is obviously not disadvantageous for the human colon despite the fact that Gb4Cer represents a perfect receptor GSL for Stx2e. Anyway, the low and high content of Gb3Cer and Gb4Cer, respectively, may explain this slight difference in cytotoxicity of Stx1a/Stx2a and Stx2e towards pHCoEpiCs observed in this study.

DRMs are widely used as model membranes or analogs for membrane microdomains resembling the liquid-ordered membrane phase and can readily be isolated showing many properties expected of lipid rafts [51,73]. It is known that DRMs are different to native lipid rafts due to their preparation using detergents. However, the application of DRMs is advantageous regarding investigations where lipid rafts are involved in certain cellular processes. Clustering of cholesterol, sphingomyelin and GSLs in microdomains is the biophysical basis for the stability of lipid rafts towards detergent-mediated disintegration [74,75]. Lipid rafts are forming portals that are targeted as attachment sites for hijacking pathogens and used as a gateway for microorganisms [76,77,78]. Microdomain-associated GSLs in the outer leaflet of the plasma membrane are appropriate recognition and attachment structures for a number of toxins as well including bacterial AB_5_ toxins [79,80]. The association of Stx with DRMs has been reported as essential requirement for a cytotoxic effect [46,52]. Here we provide the first report on the existence and distribution of Gb3Cer and Gb4Cer in DRM- and nonDRM-preparations of primary human colon epithelial cells (pHCoEpiCs) used as models for the liquid-ordered and liquid-disordered membrane phase, respectively. Both GSLs co-distributed with cholesterol and sphingomyelin preferentially in the DRM fractions suggesting lipid raft association. Low-serum cultivation with 2.5% fetal bovine serum as growth stimulating supplement and serum-free propagation of pHCoEpiCs revealed identical Gb3Cer and Gb4Cer spectra in the respective DRM preparations indicating that an influence of serum in low concentration on the cellular GSL profiles can be excluded. This finding was the basis for low-serum cultivation of the cells for further investigations beyond the initial structural proof of Stx GSL receptors performed in this study. Spectra of total GSLs and GSLs from DRM preparations showed the characteristic double bands of Gb3Cer and Gb4Cer, which have been detected in previous analyses of two types of primary human kidney epithelial cells, videlicet renal cortical (pHRCEpiCs) and proximal tubular epithelial cells (pHPTEpiCs) [28,29]. Respective upper bands harbor the Gb3Cer and Gb4Cer lipoforms with C24 long fatty acyl chains, whereas the lower ones contain the GSL species with C16 short fatty acyl chain. The biological function of this heterogeneity is so far unclear and remains to be resolved in the future. However, it is tempting to speculate that the C24:2, C24:1 and/or C24:0 long-chain fatty acyl chains of Gb3Cer and Gb4Cer may interdigitate with lipids in the inner leaflet of the plasma membrane. Such interleaflet coupling between the fatty acyl groups has been described for the C24 long fatty acyl chain of sphingomyelin in the outer leaflet of the plasma membrane with the glycerophospholipid phosphatidylserine (18:0/18:1) in the inner leaflet of the plasma membrane, a process which is known as “handshaking” and hypothesized as important mechanism in membrane biology, although much remains to be uncovered regarding the unique role of the interacting lipids [81,82]. 

New developments in matrix-assisted laser desorption/ionization mass spectrometry imaging (MALDI-MSI) with laser-induced postionization (MALDI-2-MSI) open novel innovative application options for marker-free detection of GSLs in tissue sections and cell cultures [83]. This technology has increased the ion yields for numerous lipid classes including GSLs by up to two orders of magnitude and allows sensitive MALDI MS imaging with a lateral resolution in the low micrometer range. Possible limitation by ion suppression effects due to abundant phosphatidylcholines can be overcome by on-tissue treatment with phospholipase C as shown for visualization of Stx-receptor Gb3Cer in kidney sections [84]. The simultaneous visualization of the distribution of several Gb3Cer/Gb4Cer lipoforms and those of related GSLs (e.g., precursor GSLs) by MALDI-2-MSI in tissue sections combined with immunohistochemistry has demonstrated the power of this technology [85]. Its application for in vitro cell cultures opens new avenues in “molecular histology” of individual GSL lipoforms including GSLs with singly, doubly or polyunsaturated GSLs [86], thereby improving our understanding of the GSL ceramide heterogeneity and the GSL metabolism in general.

## 4. Materials and Methods

The used materials and applied methods have been described in detail in a number of previous reports. Thus, we will provide brief descriptions in the following sections and cite the relevant references.

### 4.1. Breeding of Primary Human Colon Epithelial Cells (pHCoEpiCs)

Primary human colon epithelial cells (pHCoEpiCs) were obtained from ScienCell^TM^ (Carlsbad, CA, USA; Cat. No. 2950). The lower case “p” indicates the origin of the cells from human colon in order to distinguish these “primary” cells from genetically immortalized or tumor-derived colon epithelial cell lines. Upon receipt, cells were seeded and propagated to a sufficient amount of cells, which were then used for establishing a master cell bank of pHCoEpiCs in the 4th passage. For the pending experiments, cells were thawed and cultured in a humidified atmosphere (37 °C, 5% CO_2_) in special Colonic Epithelial Cell Medium (CoEpiCM; ScienCell^TM^, Cat. No. 2951). The colonic cells were cultured either in cell medium supplemented with fetal bovine serum (FBS) under low-serum conditions with 2.5% FBS (*v*/*v*; ScienCell^TM^, Cat. No. 0010) or without any serum under serum-free conditions and without antibiotics. Reference Vero-B4 cells, purchased from the German Collection of Microorganisms and Cell Cultures (DSMZ, Braunschweig, Germany; DSMZ no. ACC 33), were cultivated in serum-free OptiPRO^TM^ SFM medium (Gibco Life Technologies Corporation, Paisley, UK; catalogue no. 12309-019) supplemented with 4 mM l-glutamine. Cell passaging, microsocopic control and recording of the cell morphology as well as data documentation were performed following previously published standard protocols [28,29,87,88]. 

### 4.2. Treatment of pHCoEpiCs with Stx and Cytotoxicity Assay 

The effects upon exposure of pHCoEpiCs to Stx were probed with the crystal violet assay for ascertaining the cell viability, a procedure which has been previously described in several publications [28,29,39,89]. In short, cells were incubated with increasing concentrations of affinity-purified Stx1a, Stx2a or Stx2e ranging from 1 fg/mL up to 1 µg/mL. The cell viability was determined via crystal violet staining and densitometric quantification as outlined in previous publications [28,29,39,89]. The obtained data are presented as percentages related to control cells without toxin treatment corresponding to a cell viability of 100%.

### 4.3. Preparation of DRM and nonDRM Fractions

DRM and nonDRM fractions were prepared from sucrose density gradients after ultracentrifugation according to the original procedure [73,90], which was slightly modified as previously explained [28,29,87,88,91,92]. Summarized in a few words, the cell layers were disrupted with the appropriate cell lysis buffer and the cell debris was removed by gentle centrifugation (400× *g*). Short-time ultracentrifugation (150,000× *g*) was performed with the supernatant harbouring the cell membranes. The sediment was then thoroughly resolved in 1% Triton X-100 buffer and extensively mixed with an equal volume of 85% sucrose. The resulting 42.5% sucrose solution was gradually overlayed with 30% and 5% sucrose solution. The subsequent ultracentrifugation (200,000× *g*) yielded three DRM top fractions (F1 to F3) and five nonDRM fractions, further subdivided into three lower nonDRM fractions (F4 to F6, intermediate) and the two lowest nonDRM fractions (F7 to F8, bottom). These eight samples were taken step by step from the top to the bottom of the gradient followed by analysis of their lipid composition (see the next sections). 

### 4.4. Lipid Extraction and Isolation of Glycosphingolipids from Total Cells

Lipid extraction of pHCoEpiCs was conducted in the same way as previously described by us for primary human brain and kidney endothelial and primary human kidney epithelial cells [28,29,87,88]. Briefly, after methanol and chloroform/methanol extractions, alkali-labile glycerophospholipids and triglycerides were saponified by 1 M methanolic NaOH, which was carefully neutralized drop by drop with 10 M HCl. The produced NaCl and other small molecules were removed by dialysis, followed by freeze drying and solvation of the dry extract in chloroform/methanol/water (30/60/8, *v/v/v*). The neutral GSLs were isolated by anion-exchange chromatography using a small column packed with DEAE-Sepharose CL-6B (GE Healthcare, Munich, Germany) as described in earlier times [93]. Purified neutral GSLs were taken up in chloroform/methanol (2/1, *v/v*) and stored at −20 °C in glass screw cap tubes with Teflon seals. 

### 4.5. Analysis of Phospholipids and Glycosphingolipids in DRM and nonDRM Fractions

Although published several times in articles connected to the current subject, a brief explanation is given here [28,29,39,67,91,94]. Briefly summarized, gradient fractions F1 to F8 were freed from sucrose by dialysis and 90% of the fractions’ volumes were submitted to lyophilization. The dry samples were dissolved in an appropriate volume of chloroform/methanol (2/1, *v*/*v*) corresponding to 1 × 10^5^ cells/µL and used for subsequent phospholipid analysis. The remaining 10% of the dialyzed gradient fractions were employed for GSL and cholesterol analysis. After lyophilisation, mild saponification of glycerophospholipids and triglycereide with 1 M methanolic NaOH, cautious neutralization with 10 M HCl, dialysis and lyophilisation, the samples were taken up in chloroform/methanol (2/1, *v*/*v*) in a concentration equal to 1 × 10^5^ cells/µL.

### 4.6. Affinity-Purified Stx Subtypes, Antibodies and Reference Lipid Mixtures 

The renamed human-pathogenic Stx-subtypes Stx1a and Stx2a (formerly known as Stx1 and Stx2; for details refer to the modified designation of Scheutz and collaborators [95]) and the swine-pathogenic Stx2e were affinity-purified using Gb3-decorated magnetic beads. Stx1a and Stx2a were isolated from supernatants of bacterial liquid cultures of *E. coli* wild-type strains 2074/97 (serotype O145:H−) and 03-06016 (serotype O111:H−), respectively, and Stx2e was derived from wild-type *E. coli* strain 2771/07 (serotype ONT:H−) [89]. Affinity-purification was performed using sterile filtrated supernatants and the GSL binding specificities and their cytotoxic action has been documented towards various cell types as previously described [21,28,29,92,96]. The mouse anti-Stx1 and anti-Stx2 monoclonal IgG antibodies VT109/4-E9 and VT135/6-B9, respectively, were obtained from SIFIN GmbH (Berlin, Germany). 

Well characterized polyclonal chicken anti-Gb3Cer and anti-Gb4Cer IgY antibodies were employed for overlay detection of thin-layer chromatography (TLC)-separated total GSLs isolated from pHCoEpiCs and the GSL samples obtained from the sucrose gradient fractions (see above). For details, refer to previously published protocols [28,29,39,67,91,92,94]. The laboratory details including the sometimes trickery handling of the amphipathic GSLs for application of the TLC overlay technique using anti-GSL antibodies as well as GSL-specific Stx and the appropriate anti-Stx antibodies have been recently described in great depth by us [97]. Alkaline phosphatase (AP)-linked rabbit anti-chicken IgY and AP-linked goat anti-mouse IgG antibodies (Code 303-055-033 and Code 115-055-003, both from Dianova, Hamburg, Germany) were used as secondary antibodies.

A standard GSL mixture composed of equimolar amounts of the Stx-binding GSLs Gb3Cer (Galα1-4Galβ1-4Glcβ1-1Cer) and Gb4Cer (GalNAcβ1-3Galα1-4Galβ1-4Glcβ1-1Cer) served as positive control for TLC immunodetection according to previous studies [63,91,98,99]. Cholesterol (Sigma Aldrich, Steinheim, Germany; cat. no. C8667) was used as the standard for TLC analysis of DRM and nonDRM following previous protocols [67,91,94]. 

### 4.7. Thin-Layer Chromatography, Lipid Staining and Overlay Immunodetection 

Total neutral GSLs as well as the GSL samples prepared from DRM and nonDRM fractions derived from pHCoEpiCs were separated using silica gel 60-coated high-performance (HP) TLC plates (HPTLC plates, size 10 cm × 10 cm, glass-backed, silica layer thickness 0.2 mm, no. 1.05633.0001; Merck, Darmstadt, Germany). After sample administration to the silica gel surface with the semi-automatic sample applicator Linomat 5 (CAMAG, Muttenz, Switzerland), the neutral GSLs were separated in chloroform/methanol/water (120/70/17, *v*/*v*/*v*) and stained with orcinol; cholesterol was chromatographed in chloroform/acetone (96/4, *v*/*v*) and visualized with manganese(II)chloride [39,92]. TLC overlay immunodetection was performed using anti-Gb3Cer and anti-Gb4Cer antibodies as well as Stx-subtypes Stx1a, Stx2a and Stx2e together with the corresponding anti-Stx1 or anti-Stx2 antibody as previously published [67,100,101]. Details of the overlay procedure can be taken from a very recent publication containing protocols that explain all the methodological details and trickery practical handling of GSLs using anti-GSL antibodies and the Stx1a, Stx2a and Stx2e subtypes [97]. In short, overlay assays were performed with 1:2000 diluted primary chicken IgY anti-Gb3Cer and anti-Gb4Cer antibodies or with solutions of affinity-purified Stx1a, Stx2a, or Stx2e (each 0.33 µg/mL). The secondary AP-linked anti-chicken IgY antibody (1:2000 dilution) was used for detection of bound primary antibodies. Anti-Stx1 and anti-Stx2 antibodies (each 1:2000 diluted) in conjunction with AP-conjugated goat anti-mouse IgG antibody (1:2000 dilution) were used for visualization of GSL-bound Stx. 5-bromo-4-chloro-3-indolyl phosphate *p*-toluidine salt (BCIP, Roth, Karlsruhe, Germany), 0.05% (*w*/*v*) dissolved in glycine solution (pH 10.4), served as the substrate for colour development of AP-conjugated secondary antibodies yielding a blue precipitate at sites of bound anti-GSL antibodies or Stx on the silica gel layer of the TLC plate.

### 4.8. Electrospray Ionization Mass Spectrometry of Glycosphingolipids and Phospholipids

Nano electrospray ionization mass spectrometry (nanoESI MS) of GSLs and phospholipids was conducted on a SYNAPT G2-S mass spectrometer (Waters, Manchester, UK) equipped with a Z-spray source as previously described [28,29,96,102,103]. In brief, spectra were recorded in the positive ion mode with source settings as follows: temperature 80 °C, capillary voltage 0.8 kV, sampling cone voltage 20 V, and offset voltage 50 V. MS^1^ postulated GSL and phospholipid structures were confirmed by MS^2^ analysis using collision-induced dissociation (CID). After selection in the quadrupole analyser, precursor ions were separated by ion mobility with the following parameters: wave velocity 700–800 m/s, wave height 40 V, nitrogen gas flow rate 90 mL/min and helium gas flow rate 180 mL/min. Collision energies of 70 to 100 eV (E_lab_) applied to the transfer cell yielded fragment ions, which were assigned according to the nomenclature introduced by Domon and Costello for the structural characterization of GSLs [104,105].

## 5. Conclusions

So far, almost nothing was known about the exact GSL profile of primary epithelial cells from human colons, in particular the Stx receptor GSLs. With this article, we filled this gap of knowledge providing the precise structures of the various lipoforms of the Stx receptors Gb3Cer and Gb4Cer and their distribution to DRMs used as microdomain analogs of primary human colon epithelial cells. Collectively, the substantial Stx refractiveness of the cells that correlated with their low cellular content of the high-affinity Stx-receptor Gb3Cer may exclude the human colon epithelium as a major target of Stx.

## Figures and Tables

**Table 1:** Stx receptor GSLs of serum-free cultivated pHCoEpiCs determined by mass spectrometry combined with TLC immunodetection ^1^.

Compound ^2^	Ceramide	Formula	*m/z*_exp_ ^3^	*m/z*_calc_ ^3^
Gb3Cer	d18:1, C16:0	C_52_H_97_NO_18_Na	1046.67	1046.6603
Gb3Cer	d18:1, C24:1	C_60_H_111_NO_18_Na	1156.77	1156.7699
Gb3Cer *	d18:1, C24:0	C_60_H_113_NO_18_Na	1158.78	1158.7855
Gb4Cer	d18:1, C16:0	C_60_H_110_N_2_O_23_Na	1249.74	1249.7397
Gb4Cer	d18:1, C22:1	C_66_H_120_N_2_O_23_Na	1331.82	1331.8180
Gb4Cer	d18:1, C22:0	C_66_H_122_N_2_O_23_Na	1333.84	1333.8336
Gb4Cer	d18:1, C24:2	C_68_H_122_N_2_O_23_Na	1357.83	1357.8336
Gb4Cer	d18:1, C24:1	C_68_H_124_N_2_O_23_Na	1359.85	1359.8493
Gb4Cer *	d18:1, C24:0	C_68_H_126_N_2_O_23_Na	1361.87	1361.8649

^1^ All GSLs were detected in the positive ion mode as monosodiated [M+Na]^+^ species (see Figure 2, Figure 3 and Figure 4); TLC overlay detection of Gb3Cer and Gb4Cer was performed with anti-Gb3Cer and anti-Gb4Cer antibodies as well as Stx1a, Stx2a and Stx2e (see Figure 1); MS^2^ spectra of proposed Gb3Cer (d18:1, C16:0) and Gb3Cer (d18:1, C24:1) are shown in Figure 3A and Figure 3B, respectively; MS^2^ spectra of proposed Gb4Cer (d18:1, C16:0) and Gb4Cer (d18:1, C24:2/C24:1) are shown in Figure 4A and Figure 4B, respectively; ^2^ the GSLs carrying a saturated C24:0 fatty acid in the ceramide moiety are marked with an asterisk; these lipoforms were exclusively detected in the DRM F2 fractions of pHCoEpiCs (see Figures 7 and 8; ^3^ exp, experimental; calc, calculated.

## Data Availability

Data produced throughout the study are available from the corresponding author upon reasonable request.

## Supplementary Materials

The following are available online at https://www.mdpi.com/article/10.3390/ijms221810002/s1.
