# Supplementary material for: Primary Human Colon Epithelial Cells (pHCoEpiCs) Do Express the Shiga Toxin (Stx) Receptor Glycosphingolipids Gb3Cer and Gb4Cer and Are Largely Refractory but Not Resistant towards Stx
Source: Int J Mol Sci. 2021 Sep 16;22(18):10002. doi: 10.3390/ijms221810002 (PMC8472147; doi:10.3390/ijms221810002)
Supplement: Supplementary file 1 [file ijms-22-10002-s001.zip › ijms-1374316-supplementary.pdf]

Supplementary Materials:

Primary Human Colon Epithelial Cells (pHCoEpiCs) Do Express the Shiga Toxin (Stx) Receptor Glycosphingolipids Gb3Cer and Gb4Cer and Are Largely Refractory but not Resistant towards Stx

Johanna Detzner[1], Charlotte Püttmann[1], Gottfried Pohlentz[1], Hans-Ulrich Humpf[2], Alexander Mellmann[1], Helge Karch[1] and Johannes Müthing [1,*]

[1] Institute for Hygiene, University of Münster, 48149 Münster, Germany; Johanna.Detzner@ukmuenster.de (JD), lotte02021996@web.de (CP), pohlentz@uni-muenster.de (GP), Alexander.Mellmann@ukmuenster.de (AM), Helge.Karch@ukmuenster.de (HK), jm@uni-muenster.de (JM)

[2] Institute for Food Chemistry, University of Münster, 48149 Münster, Germany; humpf@uni-muenster.de (HUH)

* Correspondence: jm@uni-muenster.de; Tel.: +49-(0)251-8355192

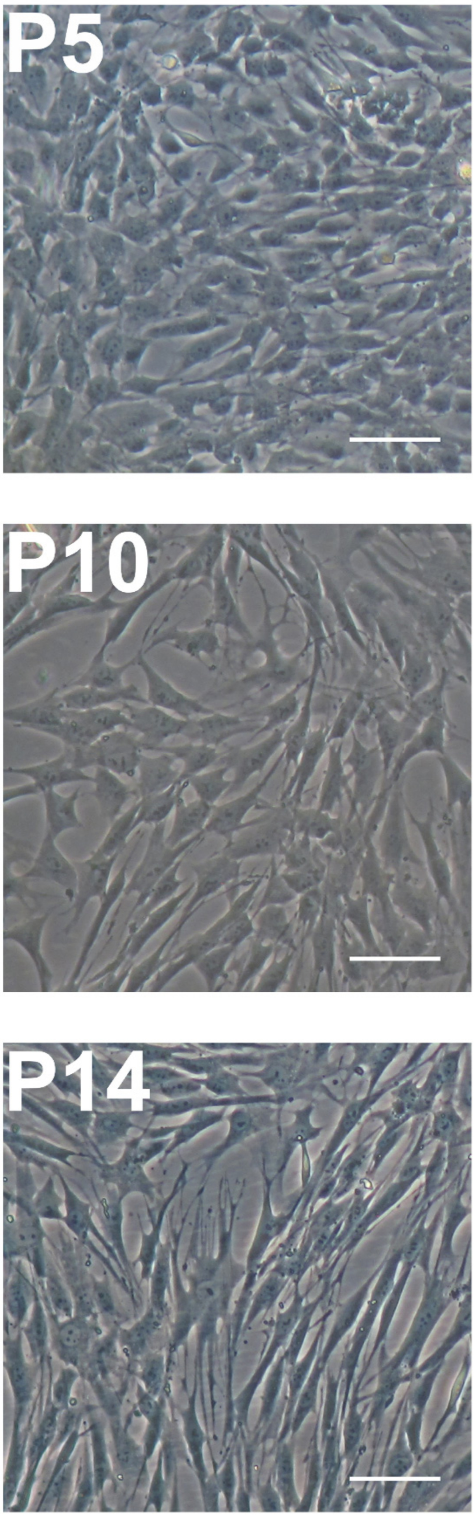

Figure S1. Light microscopy micrographs of pHCoEpiCs during passage 5 (P5), passage 10 (P10), and passage 14 (P14) at approximate 90% confluence. Original magnification x10. Bar: 100 µm

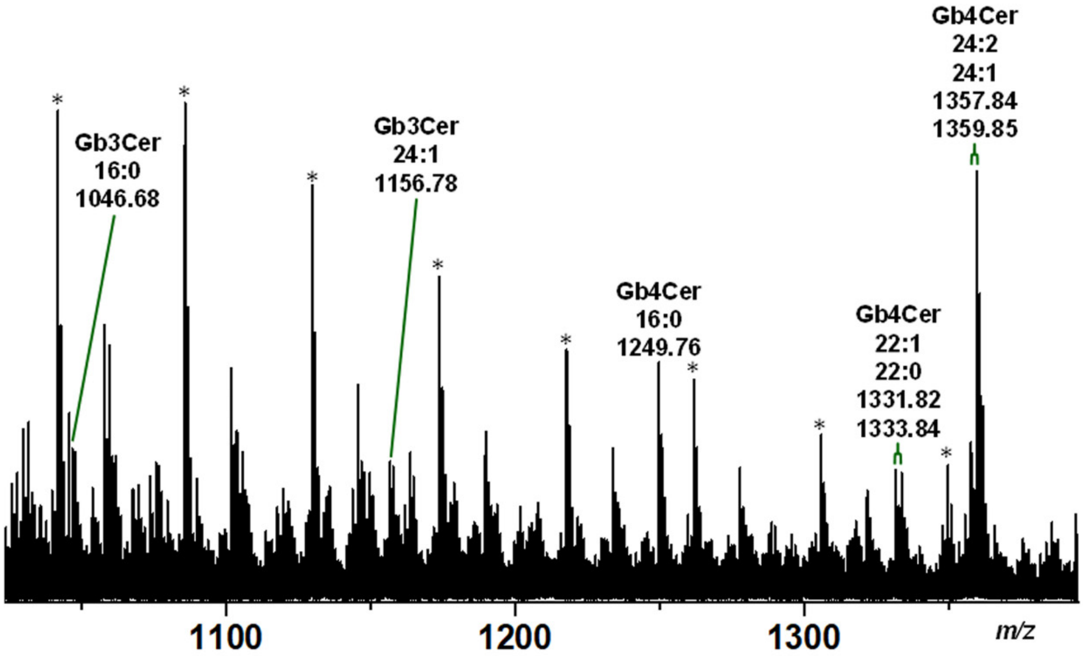

Figure S2. Overview MS[1] spectrum of Stx receptor GSLs Gb3Cer and Gb4Cer of serum-free cultivated pHCoEpiCs. The spectrum was obtained from a GSL preparation of replicate 1 (R1; see Figure 1) showing the various lipoforms of Gb3Cer and Gb4Cer harboring sphingosine (d18:1) as the sole sphingoid base and variable fatty acids as indicated. All GSLs were detected as monosodiated species using the positive ion mode. The asterisks mark polyethylene glycols (PEGs) appearing as serial contaminations in the GSL preparation.

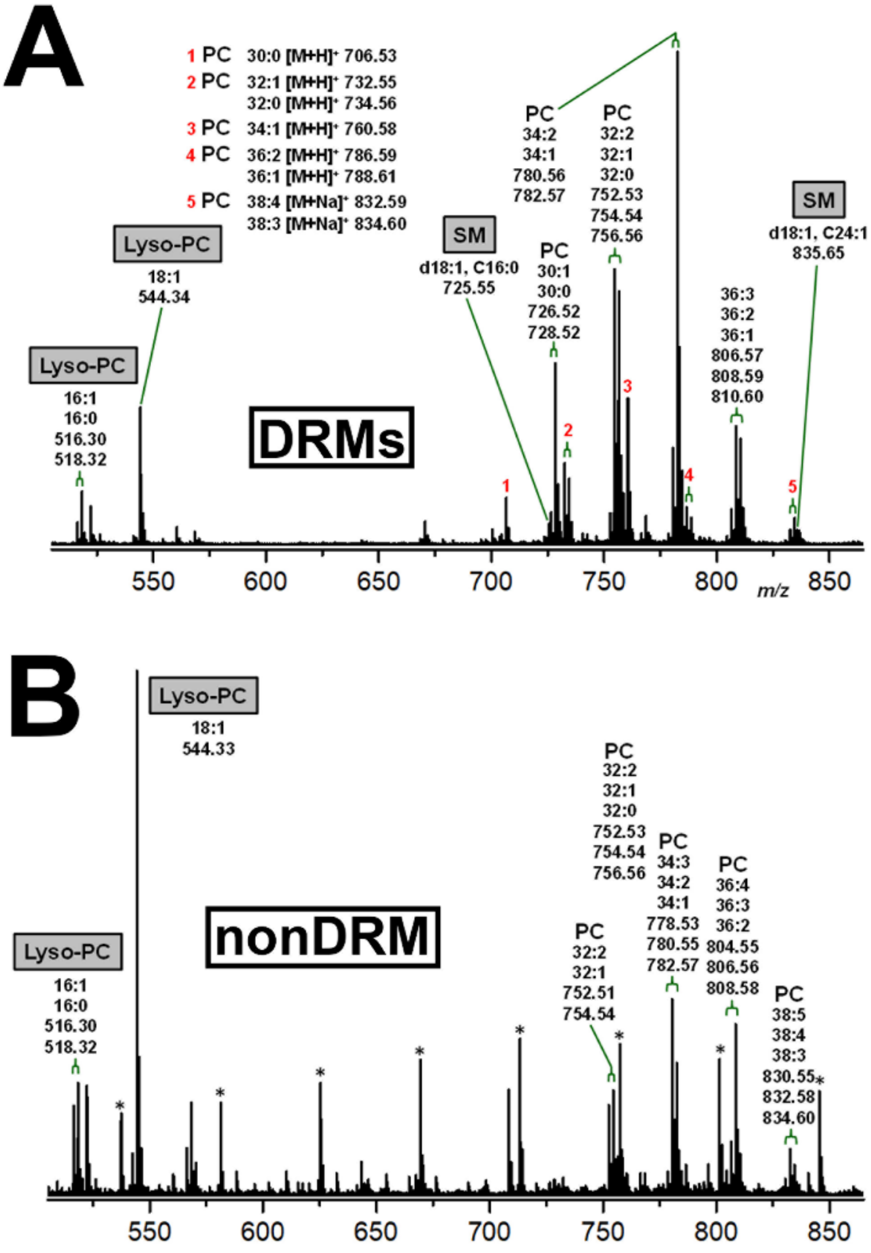

Figure S3. Phospholipid MS[1] spectra of DRM (**A**) and nonDRM fractions (**B**) derived from low-serum cultivated pHCoEpiCs. MS spectra of combined lipid extracts from DRM F2 and F3 and nonDRM F7 fractions of replicate 2 obtained in the positive ion mode are exemplarily shown. Unless otherwise stated sodiated species ($[M+Na]^+$) detected are assigned. The SM species occurring only in DRMs are considered as markers of the liquid-ordered membrane phase and highlighted as grey boxes. The lyso-PCs in the nonDRM fraction can be considered as markers for the liquid-disordered membrane phase due to their exorbitant content in the nonDRM fraction F7 and are framed in grey boxes as well. The asterisks mark polyethylene glycols (PEGs) appearing as serial contaminations in the lipid preparation.

Table S1. Relative content of Gb3Cer, Gb4Cer, and cholesterol in sucrose gradient fractions obtained from pHCoEpiCs.

Gb3Cer.

Fraction	Replicate 1 [%]	Replicate 2 [%]
F1 DRM (top)	0.0	0.0
F2 DRM (top)	51.0	57.3

F3 DRM (top)	5.7	Σ56.7 (F1-F3)	22.9	Σ80.2 (F1-F3)
F4 nonDRM (intermediate)	0.0		0.0	
F5 nonDRM (intermediate)	0.0		0.0	
F6 nonDRM (intermediate)	7.4	Σ7.4 (F4-F6)	0.0	Σ0.0 (F4-F6)
F7 nonDRM (bottom)	20.2		19.8	
F8 nonDRM (bottom)	15.7	Σ35.9 (F7-F8)	0.0	Σ19.8 (F7-F8)
	Σ100.0 (F1-F8)		Σ100.0 (F1-F8)	

Gb4Cer.

Fraction	Replicate 1 [%]		Replicate 2 [%]	
F1 DRM (top)	0.0		0.0	
F2 DRM (top)	51.0		51.5	
F3 DRM (top)	13.4	Σ64.4 (F1-F3)	27.9	Σ79.4 (F1-F3)
F4 nonDRM (intermediate)	0.0		0.0	
F5 nonDRM (intermediate)	0.0		3.2	
F6 nonDRM (intermediate)	6.4	Σ6.4 (F4-F6)	0.0	Σ3.2 (F4-F6)
F7 nonDRM (bottom)	19.7		17.4	
F8 nonDRM (bottom)	9.5	Σ29.2 (F7-F8)	0.0	Σ17.4 (F7-F8)
	Σ100.0 (F1-F8)		Σ100.0 (F1-F8)	

Cholesterol.

Fraction	Replicate 1 [%]		Replicate 2 [%]	
F1 DRM (top)	0.0		0.0	
F2 DRM (top)	32.5		46.0	
F3 DRM (top)	11.2	Σ43.7 (F1-F3)	23.1	Σ69.1 (F1-F3)
F4 nonDRM (intermediate)	2.7		1.7	
F5 nonDRM (intermediate)	2.6		3.9	
F6 nonDRM (intermediate)	10.3	Σ15.6 (F4-F6)	1.5	Σ7.1 (F4-F6)
F7 nonDRM (bottom)	21.2		23.8	
F8 nonDRM (bottom)	19.5	Σ40.7 (F7-F8)	0.0	Σ23.8 (F7-F8)
	Σ100.0 (F1-F8)		Σ100.0 (F1-F8)	
